# P-838. T2Candida PCR Positivity Without Blood Culture Confirmation: Does It Matter?

**DOI:** 10.1093/ofid/ofaf695.1046

**Published:** 2026-01-11

**Authors:** Vishak Hari Kumar, Ali Ibrahim, Daniel Li, Joyce Xu, Navaneeth Narayanan, Tanaya Bhowmick, John P Mills, Thomas Kirn, Keith S Kaye, Ahmed Abdul Azim

**Affiliations:** Rutgers Robert Wood Johnson University Hospital, Holland, Pennsylvania; Rutgers - New Brunswick, Edison, New Jersey; RWJMS, South Amboy, NJ; Rutgers Robert Wood Johnson Medical School, New Brunswick, New Jersey; Rutgers University Ernest Mario School of Pharmacy & Robert Wood Johnson University Hospital, New Brunswick, NJ; Rutgers Robert Wood Johnson Medical School, New Brunswick, New Jersey; Rutgers Robert Wood Johnson Medical School, New Brunswick, New Jersey; Rutgers Robert Wood Johnson Medical School, New Brunswick, New Jersey; Rutgers Robert Wood Johnson Medical School, New Brunswick, New Jersey; Rutgers Robert Wood Johnson Medical School, New Brunswick, New Jersey

## Abstract

**Background:**

Invasive candidiasis remains a major cause of morbidity and mortality in hospitalized patients. The T2Candida® (T2) system (T2 Biosystems®, Lexington, MA, USA), a Nucleic acid-based diagnostic test, demonstrates high sensitivity for detecting five Candida species compared to conventional blood cultures (BC). However, the clinical significance of discordant results (T2 positive/BC negative) remains uncertain. We evaluated clinical outcomes among adult inpatients with concordant and discordant T2 and BC results.

**Methods:**

We conducted a single-center retrospective analysis of hospitalized adults with T2Candida-positive tests from June 2022 to December 2024. Patients were classified as T2 positive/BC positive (concordant) and T2 positive/BC negative (discordant). Demographics, comorbidities, antimicrobial therapy, and clinical outcomes were collected through administrative data and manual chart reviews. Composite adverse outcomes were defined as any metastatic infection, relapse within 90 days, or mortality within 30 days. Chi-square and t-test were used to compare variables.

**Results:**

Among 84 T2-positive patients, 23 were concordant and 61 were discordant (Table 1). The most common T2 result was *C. albicans/C. tropicalis* in both groups, 14/23 (60.9%) in the concordant group and 31/61 (50.8%) in the discordant group (Table 2). Baseline demographics, comorbidities, and immunocompromised status were similar across groups. Notably, 33% of the entire cohort was immunocompromised. Effective antifungal therapy was administered consistently across groups, except in four cases: three early deaths and one result deemed a contaminant. 30-day mortality occurred in 39 of 84 patients (46.4%): 12/23 (52.2%) in the concordant group, and 27/61 (44.3%) in the discordant group (p = 0.687). Although adverse outcomes were numerically worse in the concordant group, there were no statistically significant differences in 14-day mortality, 30-day mortality, metastatic infection, relapse, or composite adverse outcomes between groups (Table 3).

**Conclusion:**

While our findings suggest that clinical outcomes are comparable between T2 positive/BC negative and T2 positive/BC positive patients, further studies with larger sample sizes are needed to validate these results.

**Disclosures:**

Thomas Kirn, MD PhD, BD: Advisor/Consultant|BD: Honoraria Keith S. Kaye, MD, MPH, AbbVie: Advisor/Consultant|GSK: Advisor/Consultant|Merck: Advisor/Consultant|Shionogi: Advisor/Consultant

